# Cell banking for regulatory T cell-based therapy: strategies to overcome the impact of cryopreservation on the Treg viability and phenotype

**DOI:** 10.18632/oncotarget.23887

**Published:** 2018-01-03

**Authors:** Karolina Gołąb, Randall Grose, Veronica Placencia, Amittha Wickrema, Julia Solomina, Martin Tibudan, Evelyn Konsur, Kamil Ciepły, Natalia Marek-Trzonkowska, Piotr Trzonkowski, J. Michael Millis, John Fung, Piotr Witkowski

**Affiliations:** ^1^ Department of Surgery, University of Chicago, Chicago, IL, USA; ^2^ South Australian Health and Medical Research Institute, University of Adelaide, SA, Australia; ^3^ Department of Medicine, Hematology-Oncology, Cancer Research Center, University of Chicago, Chicago, IL, USA; ^4^ Department of Family Medicine, Medical University of Gdańsk, Gdańsk, Poland; ^5^ Department of Clinical Immunology and Transplantology, Medical University of Gdańsk, Gdańsk, Poland

**Keywords:** regulatory T cells (Tregs), cell banking strategies, effect of cryopreservation, Treg-based therapies

## Abstract

The first clinical trials with adoptive Treg therapy have shown safety and potential efficacy. Feasibility of such therapy could be improved if cells are cryopreserved and stored until optimal timing for infusion.

Herein, we report the evaluation of two cell-banking strategies for Treg therapy: 1) cryopreservation of CD4^+^ cells for subsequent Treg isolation/expansion and 2) cryopreservation of *ex-vivo* expanded Tregs (CD4^+^CD25^hi^CD127^lo/-^ cells). First, we checked how cryopreservation affects cell viability and Treg markers expression. Then, we performed Treg isolation/expansion with the final products release testing.

We observed substantial decrease in cell number recovery after thawing and overnight culture. This observation might be explained by the high percentage of necrotic and apoptotic cells found just after thawing. Furthermore, we noticed fluctuations in percentage of CD4^+^CD25^hi^CD127^-^ and CD4^+^FoxP3^+^ cells obtained from cryopreserved CD4^+^ as well as Treg cells. However, after re-stimulation Tregs expanded well, presented a stable phenotype and fulfilled the release criteria at the end of expansions.

Cryopreservation of CD4^+^ cells for subsequent Treg isolation/expansion and cryopreservation of expanded Tregs with re-stimulation and expansion after thawing, are promising solutions to overcome detrimental effects of cryopreservation. Both of these cell-banking strategies for Treg therapy can be applied when designing new clinical trials.

## INTRODUCTION

T regulatory cells (Tregs) are characterized by expression of CD4 and CD25 surface markers and play critical roles in maintaining the status quo of the immune system by hampering activation and proliferation of responder T cells [1]. Since the discovery of this cell population there has been an increasing interest in utilization of their properties for clinical application in the treatment of autoimmune diseases and immunomodulation in transplantation. Animal studies presented success of Treg application [2–11], which encouraged investigators to initiate clinical trials utilizing Tregs. Several phase 1 trials have already indicated that the therapy is safe and efficacious [12–17]. Better identification of Tregs with transcription factor forkhead box P3 (FoxP3), which regulates Treg development and function [18–20], accelerated clinical Treg application. It was also established that expression of CD127 surface marker inversely correlated well with FoxP3^+^ [21], which triggered development of techniques allowing *ex-vivo* human Treg isolation and expansion [22–28]. Subsequently, clinical trials emerged testing different clinical Treg approaches in autoimmune diseases [29], liver transplantation [26] and kidney transplantation (“The ONE Study” [30] and “TASK” [31]). Optimal Treg dose and timing of the application as well as supportive pharmacological therapy have yet to be determined [32].

From a logistical perspective, it would be much more convenient if pure Tregs or other cells containing Tregs could be stored in sufficient quantity, allowing Tregs to be applied at an optimal time without prolonged processing [33, 34]. In deceased renal, liver or other organ transplantation, the timing of the procedure is unpredictable and depends on donor availability. Therefore banking of cryopreserved Treg cells that are ready to be used is critically important [32]. Feasibility of such approach is currently being tested in one of the clinical studies [30, 35].

The effects of cryopreservation on the Treg cell population have not been well defined. Based on reports of freezing\thawing of Peripheral Blood Mononuclear Cells (PBMCs), cryopreservation may affect cytokine production and expression of surface markers essential for Treg function [33, 36–38]. Moreover, upon thawing, Treg viability and suppressive function can be also compromised, which may significantly affect the clinical safety and efficacy of this therapy [34, 39]. As a result, there is still a need to investigate the impact of cryopreservation on the population of human T regulatory cells to be able to define the optimal protocols for Treg cell banking.

**Figure 1:**
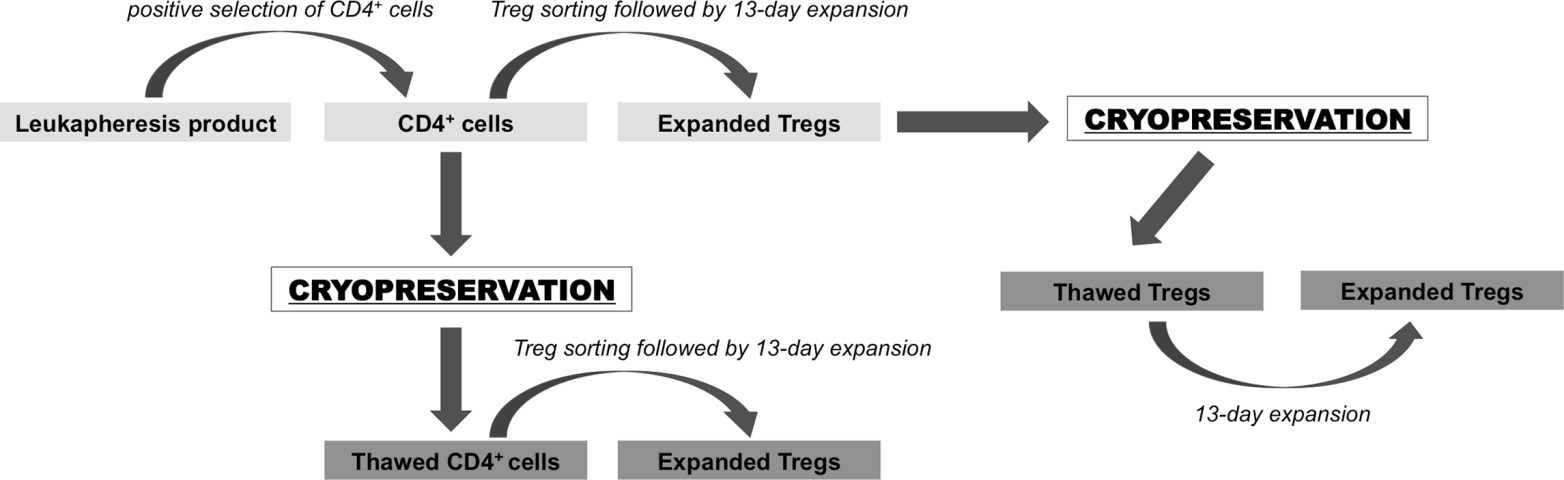
Schema of cryopreservation strategies for Treg therapy tested in the study CD4^+^ cells were pre-enriched from leukapheresis product via immunomagnetic positive selection on CliniMACS^®^ device. A portion of these cells was cryopreserved and the rest was used directly for Treg FACS isolation. Sorted Tregs were expanded *ex-vivo* for 13 days and after expansion cryopreserved. After over 1 year of storage, frozen CD4^+^ cells were thawed and used for Treg sorting and expansion. Cryopreserved Tregs were thawed and then also expanded in the same fashion as Tregs isolated from fresh frozen CD4^+^ cells.

In this study, we tested two strategies of cryopreservation and cell banking, which are both feasible to apply in the clinical setting. In the first one, we cryopreserved CD4^+^ cells isolated from the human product of leukapheresis serving as a cell source for subsequent Treg isolation and *ex vivo* expansion. In the second approach, we froze Tregs after isolation and 13-day expansion (Figure 1). Upon thawing, we analyzed cell viability and apoptosis as well as Treg phenotype to determine the effects of the cryopreservation process on those cells. Due to the low Treg cell recovery and cell marker instability in the second approach, we re-stimulated and expanded them *ex-vivo* again to assess whether they resumed their original property and high number. Importantly, all the procedures of cell isolation, cryopreservation, thawing and expansion were done accordingly to current Good Manufacturing Procedures (cGMP) in a clinical cell processing facility to confirm that the processes could be used in the clinical setting. Finally, Tregs generated in both approaches were tested to ensure fulfillment of release criteria for clinical application [28].

## RESULTS

### Poor CD4^+^ and Treg cell recovery after cryopreservation is associated with impaired cell viability

**Figure 2:**
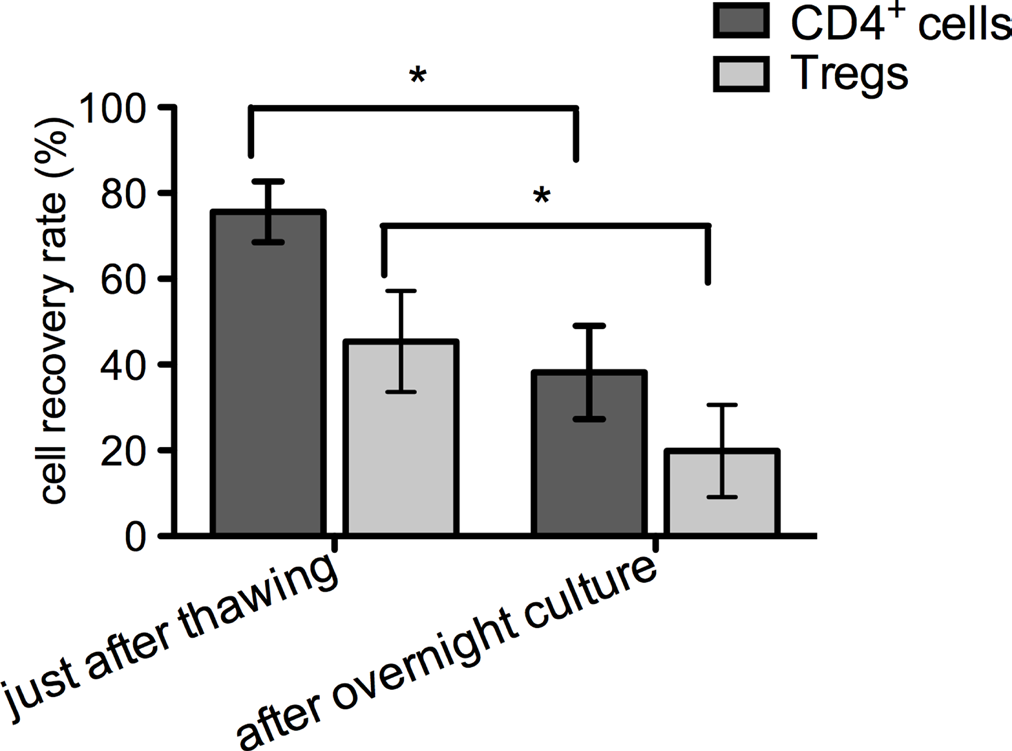
Post-thawing cell recovery rate CD4^+^ and Treg cell counting was performed as described and graph depicts the percentage of all recovered cells just after thawing and overnight culture in comparison to number of cells that were cryopreserved. Results are expressed as mean ± SD; (*n* = 3) for cryopreserved CD4^+^ cells and (*n* = 4) for cryopreserved Tregs; significance was determined by paired *t*-test; ^*^P < 0.05.

**Figure 3:**
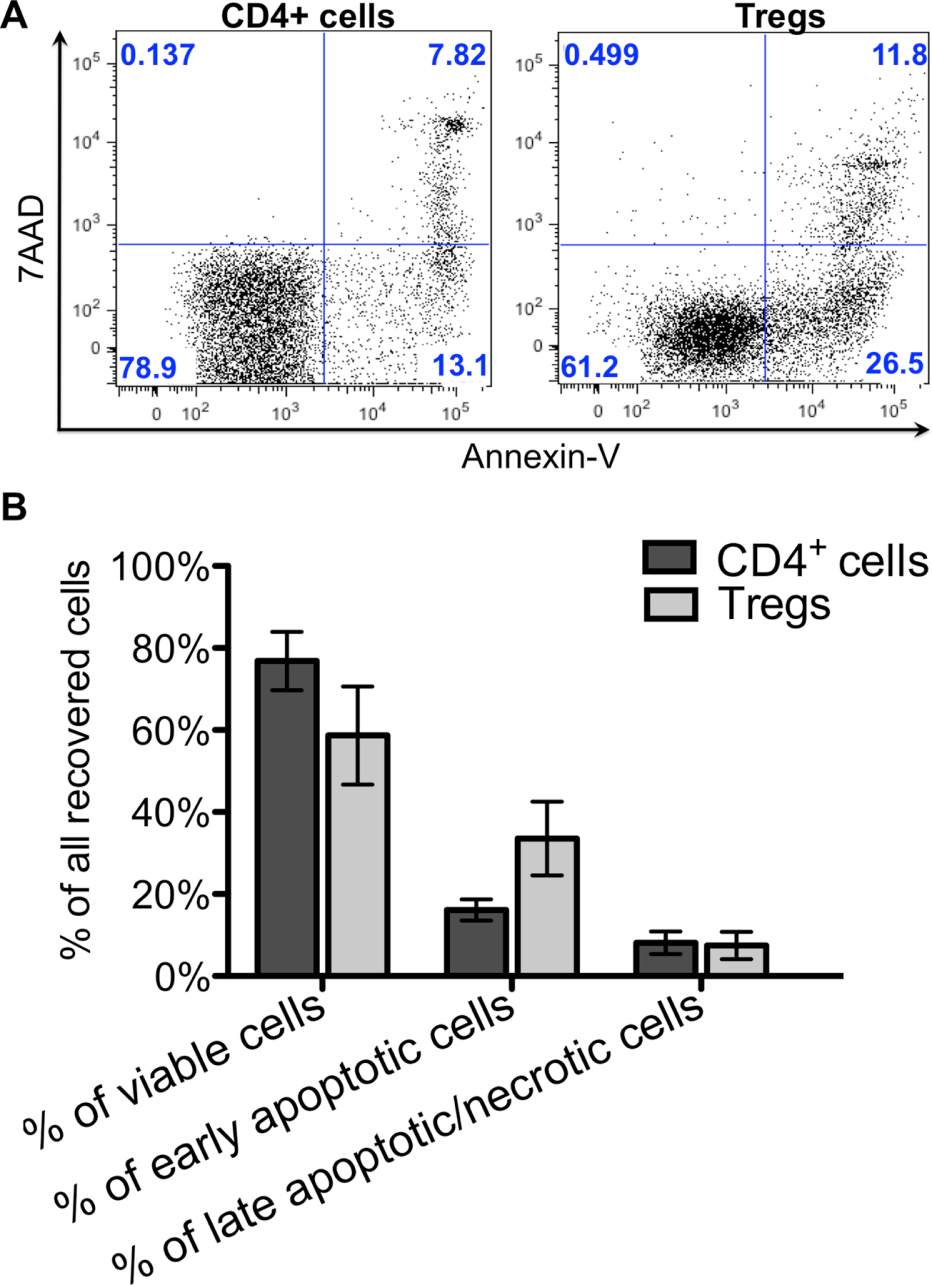
Cell viability and apoptosis just after thawing Viability with apoptosis of thawed CD4^+^ and Treg cells was analyzed after staining with Annexin V and 7AAD. (**A**) Flow cytometric dot plots from representative experiment showing percentage of viable – Annexin V^-^ 7AAD^-^, early apoptotic – Annexin V^+^ 7AAD^-^ and late apoptotic/necrotic – Annexin V^+^, 7AAD^+^ for Treg and CD4^+^ cells. (**B**) Graph showing viability with apoptosis of thawed CD4^+^ and Treg cells. Results are expressed as mean ± SD; (*n* = 3) for cryopreserved CD4^+^ cells and (*n* = 4) for cryopreserved Tregs.

The average percentage of CD4^+^ cells that recovered immediately after thawing was 75.6 ± 7.1%, however the recovery rate for cryopreserved Tregs was lower: 45.4 ± 11.8% (Figure 2). After culturing overnight, the cell numbers decreased for both CD4^+^ cells and Tregs, resulting in the final post-thaw recovery rates: 38.2 ± 10.9% and 19.9 ± 10.7%, respectively (Figure 2). Results of apoptosis assays performed immediately after thawing showed that 16.1 ± 2.6% of all CD4^+^ cells indicated early apoptosis and 8.1 ± 2.7% late apoptosis/necrosis (Figure 3). For thawed Tregs, the frequency of early apoptotic cells was 33.6 ± 9% and late apoptotic/necrotic 7.5 ± 3.3% (Figure 3). Relatively high percentage of apoptotic cells, found immediately after thawing might be responsible for subsequent cell destruction and could explain the additional loss in cell number observed after the overnight culture.

### Treg phenotype fluctuation upon cryopreservation

**Figure 4:**
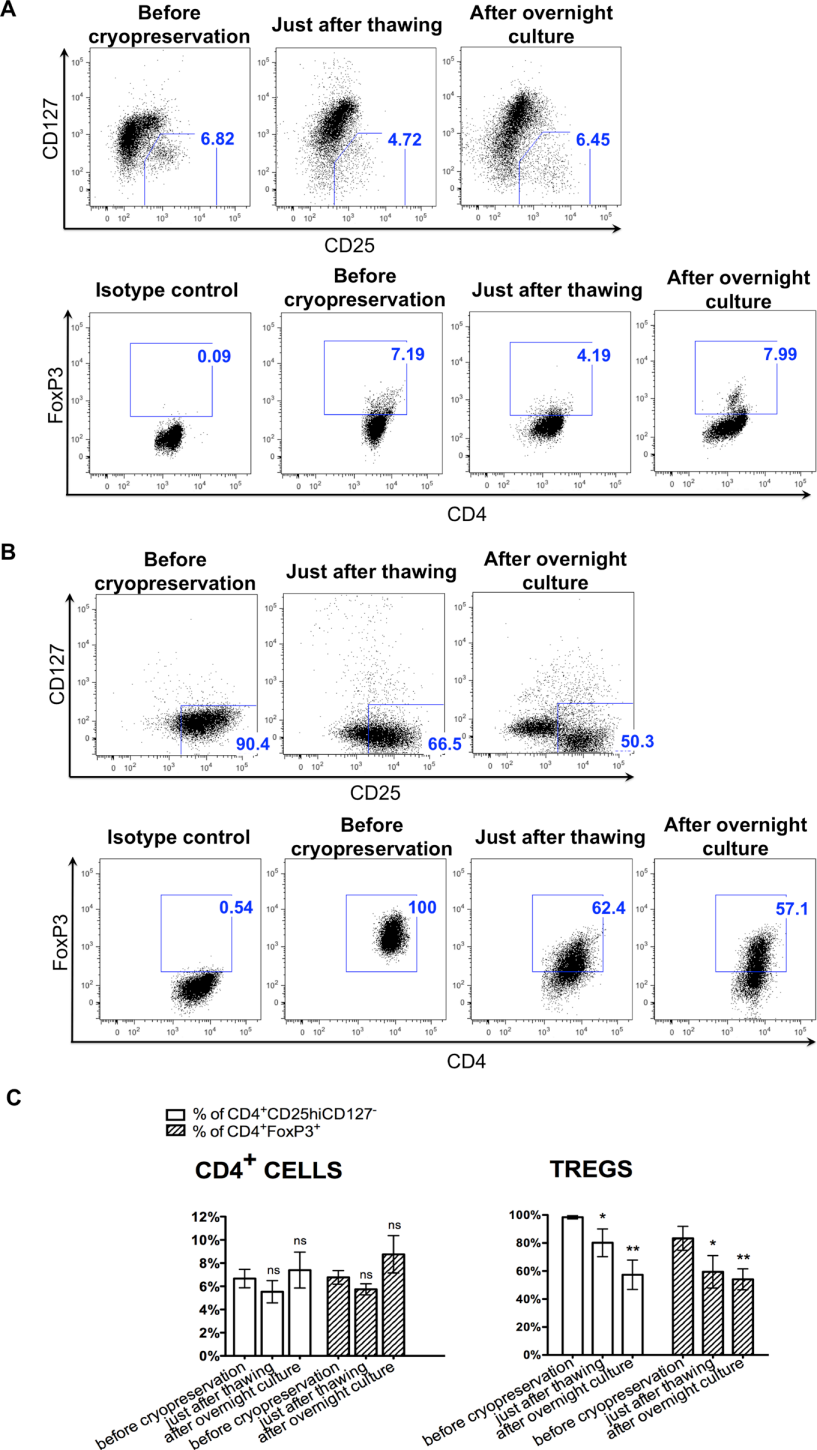
Instability of Treg phenotype upon cryopreservation Flow cytometric analysis of CD4, CD25, CD127, FoxP3 expression in CD4^+^ and Treg cells was performed before cryopreservation, just after thawing and overnight culture. (**A**, **B**) Flow cytometric dot-plots from the representative experiments showing gate for CD4^+^CD25^hi^CD127^-^ cells and CD4^+^FoxP3^+^ cells before cryopreservation, just after thawing and after overnight culture, for CD4^+^ cells and Tregs, respectively. (**C**) Graph showing changes in the percentage of CD4^+^CD25^hi^CD127^-^ cells and CD4^+^FoxP3^+^ cells before cryopreservation, just after thawing and after overnight culture for CD4^+^ and Treg cells. Results are expressed as mean ± SD; *n* = 3 for cryopreserved CD4^+^ cells and *n* = 4 for cryopreserved Tregs. The percentages of cells with phenotype CD4^+^CD25^hi^CD127^-^ and CD4^+^FoxP3^+^ just after thawing and after overnight culture were compared to these values before cryopreservation; significance was determined by paired *t*-test; ^*^P < 0.05; ^**^P < 0.005.

Based on the studies assessing Treg frequency in fresh and frozen/thawed samples of PBMCs, it is known that cryopreservation can affect specific Treg markers expression [33]. Therefore, upon thawing of both isolated CD4^+^ and expanded Tregs, we checked the percentage of cells with characteristic Treg phenotype: CD4^+^CD25^hi^CD127^-^ and CD4^+^FoxP3^+^. For CD4^+^ cells, we observed the following changes: the mean percentage of CD4^+^CD25^hi^CD127^-^ decreased from 6.7 ± 0.8% to 5.5 ± 7.4% and the frequency of cells with the phenotype CD4^+^FoxP3^+^ decreased from 6.7 ± 0.6% to 5.7 ± 0.5%. After an overnight culture, the proportions of these subpopulations increased to 7.4 ± 1.5% and 8.8 ± 1.6%, respectively (Figure 4A, 4C). However, these changes were not statistically significant.

When we checked the phenotype of expanded Tregs upon cryopreservation, we noticed a significant decrease in the cell rate expressing Treg phenotype. The percentage of CD4^+^CD25^hi^CD127^-^ cells decreased from 98.4 ± 1.2% to 80.2 ± 9.9% (*p* < 0.05) upon thawing and the percentage of CD4^+^FoxP3^+^ cells declined from 83.3 ± 8.5% to 59.5 ± 11.6% (*p* < 0.05; Figure 4B, 4C). After an overnight culture, further decrease in the frequency of CD4^+^CD25^hi^CD127^-^ and CD4^+^FoxP3^+^ cells among all retrieved Treg cells was observed to 57.4 ± 10.5% and 54.1 ± 7.5%, respectively (*p* < 0.005; Figure 4B, 4C).

### Tregs can be sorted from thawed CD4^+^ cells. Both thawed Tregs and Tregs isolated from cryopreserved CD4^+^ cells present stable phenotypes during the *ex-vivo* expansion

**Figure 5:**
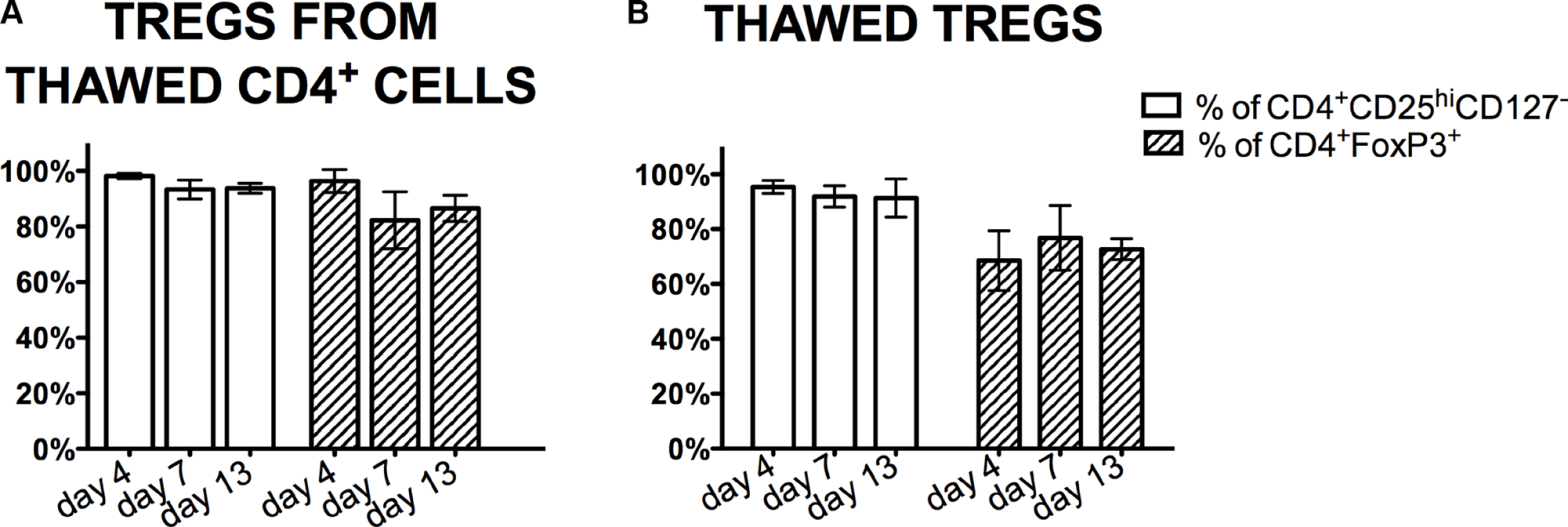
Treg phenotype during the expansion processing Analysis of Treg markers CD4, CD25, CD127, FoxP3 expression was done by flow cytometry during the expansion process of Tregs isolated from thawed CD4^+^ cells (**A**) and thawed Tregs (**B**). Results are expressed as mean ± SD; *n* = 3 for cryopreserved CD4^+^ cells and *n* = 4 for cryopreserved Tregs.

Tregs were sorted from cryopreserved CD4^+^ cells, then stimulated and expanded for 13 days. Despite poor viability and instability of characteristic marker expression upon thawing of CD4^+^ cells (as described above), we were able to sort Tregs (CD4^+^CD25^hi^CD127^-^ cells) from this population of CD4^+^ cells. The average purity of isolated Tregs was 98.3 ± 0.9% and viability of 98.0 ± 2.4%. During the expansion, they maintained stable Treg characteristic phenotype percentage of CD4^+^CD25^hi^CD127^-^ cells was > 93% and of CD4^+^FoxP3^+^ cells was > 82% at all time checkpoints (Figure 5A).

In the second set of experiments, cryopreserved Tregs after thawing were stimulated and then *ex-vivo* expanded. Despite significant decrease in the percentage of cells with the phenotype CD4^+^CD25^hi^CD127^-^ and CD4^+^FoxP3^+^ after thawing, upon re-stimulation and repeated expansion, Treg phenotype was restored and the mean percentages of CD4^+^CD25^hi^CD127^-^ and CD4^+^FoxP3^+^ cells was 95.4 ± 2.4%, 91.9 ± 3.9%, 91.3 ± 7%, 68.5 ± 10.9%, 76.8 ± 11.8%, and 72.7 ± 3.8% on day 4, 7, 13 of expansion, respectively (Figure 5B).

Both, Tregs isolated from cryopreserved CD4^+^ cells and thawed previously expanded Tregs after stimulation, expanded well with the average fold increase in cell number after 13 days of 193 ± 233.8 (range 31- 461) and 109 ± 135.4 (range 35- 312), respectively.

### Tregs isolated from cryopreserved CD4^+^ cells and thawed Tregs after expansion passed all release criteria

The FDA requires that biological product must be characterized with appropriate tests for identity, purity, safety and potency in order to be applied clinically [40]. We adopted release criteria for the final Treg clinical product from on-going clinical trials [39, 41, 42] and also applied them to our previously published clinical-grade Treg manufacturing protocol [28]. Since the aim of this work was to test cryopreservation and cell banking strategies for clinical therapy, on the last day of Treg expansions, we processed cells as final clinical Treg product, with testing for release criteria assays for clinical application. Final Treg products generated from each expansion – both after CD4^+^ cells cryopreservation and Treg cryopreservation, fulfilled all established release criteria:

- Gram stain was negative and there was no aerobic, anaerobic or fungal growth (tests performed by College of American Pathologists (CAP) CAP-certified Clinical Microbiology Laboratory)
- Endotoxin concentration was < 5 EU/kg (calculated for an average 70 kg person receiving final product; analysis done by EndoSafe PTS Endotoxin System;)
- Cells were free from mycoplasma contamination (< 0.8 in Lonza MycoAlert Mycoplasma Assay)
- There were less than 100 expansion beads per 3 × 10^6^ cells (beads were counted on hemocytometer after cell permeabilization)
- Percentage of CD4^+^ and FoxP3^+^ cells were: > 90% and > 60%, respectively
- Percentage of contaminating CD8^+^ cells was < 5%
- Viability was > 75%

Analysis of % of CD4^+^, FoxP3^+^, CD8^+^ cells and viability with 7AAD was performed by flow cytometry in CAP-certified Clinical Hematology Laboratory

### Expanded Tregs isolated from cryopreserved CD4^+^ cells and cryopreserved Tregs after re-expansion indicated good suppressive abilities

**Figure 6:**
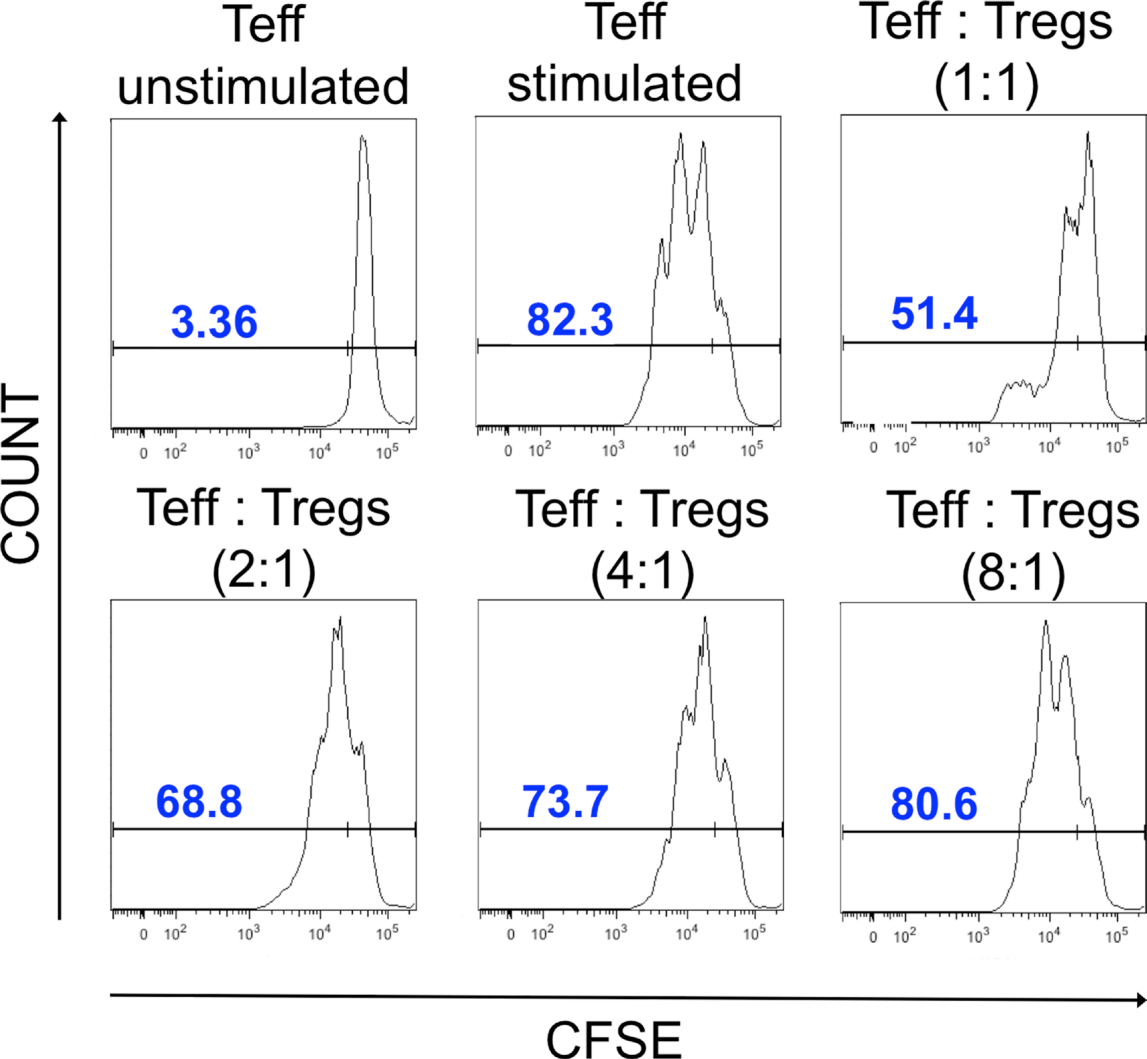
Suppressive properties of Tregs after expansion Histograms showing CFSE dilution in unstimulated and stimulated Teffectors and stimulated Teffectors co-cultured in presence of Tregs at different ratios. Unstimulated Teffectors show less CFSE dilution and served as negative control. In presence of Tregs, CFSE dilution was lower in comparison to stimulated Teffectors cultured without Tregs, indicating that Tregs suppress proliferation of Teffectors. The CFSE dilution was correlated with the Treg dose. The data shown are from the representative experiment.

At the end of expansions of both: Tregs isolated from cryopreserved CD4^+^ cells and Tregs re-expanded after cryopreservation, we performed a suppression of proliferation assay with CFSE-stained Teffectors to check Tregs suppressive properties. Addition of Tregs to stimulated Teffectors inhibited Teffector cell division (Figure 6). This effect was Treg-dose dependent and still measurable at the ratio Teffectors to Tregs – 8:1. There was no difference observed in suppressive properties between Tregs isolated from cryopreserved CD4^+^ cells and Tregs re-expanded after cryopreservation.

## DISCUSSION

Adoptive transfer of Tregs as a therapy could be more easily clinically applied if ready-to-use cell products could be stored to be available for infusion to the patient at the optimal time. Furthermore, cells could be manufactured in highly specialized centers and distributed for therapy to distant clinical locations. Cell banking would also allow for better synchronization of Treg application and pharmacological immunomodulation. It would allow for retrieving of cells from patient for processing prior to pharmacological therapy, which could affect Treg recovery and/or function. Additionally, such immunomodulation may further improve the effect of Tregs after cell infusion [43]. Currently, the immunosuppression used in clinical practice usually has a detrimental effect on Tregs and only few medications like mTOR inhibitors, are Treg “friendly” [43].

In this study, we focused on two main strategies that can be utilized for cell banking in Treg-based therapies: 1) cryopreservation of the cells, which could be a source material for Treg isolation and expansion and 2) cryopreservation of already isolated/ *ex-vivo* expanded Tregs (Figure 1). Many Treg manufacturing protocols involve a two-step Treg isolation process, with pre-enrichment of the CD4^+^ cell population carried out as the first step [14, 16, 26, 27, 34, 44–46]. Retrieved number of CD4^+^ cells varies and is dependent on the initial source of leukocytes. Utilizing leukapheresis as a source, we obtained over 1 billion of CD4^+^ cells and using only half of those cells for Treg Fluorescence Activated Cell Sorting (FACS) and *ex-vivo* expansion, we still obtained high enough Treg numbers for clinical application [28]. The remaining CD4^+^ cells, not used for direct sorting, could be frozen and stored for subsequent Treg isolations/expansions. In the second approach we proposed, Tregs could be cryopreserved right after *ex-vivo* expansion. This could be done with either all expanded cells or with only the portion that remained after infusion, depending on the strategy and the protocol.

Cell cryopreservation has many downsides. It can significantly affect cell physiology, receptor expression, lead to apoptosis, necrosis and cell death as was shown with PBMCs [33, 47]. On one hand, there are reports showing that Tregs can be successfully frozen and thawed without compromising their phenotype [26, 45, 48, 49]. However, we noticed a concerning fact in one of these reports that there was lack of increase in the frequency and absolute number of Tregs in the recipients’ peripheral blood after cryopreserved Treg infusion, as it was observed during initial infusion of “fresh” Tregs in the same group of patients in the clinical Graft-versus-Host-Disease (GvHD) trial [39]. It may indicate that despite presenting good viability immediately after thawing, shortly prior to the infusion, Tregs might be already altered at this time by apoptosis and die soon after infusion. Our results confirmed such possibility. Although immediately after thawing many Tregs seem to be viable, they already expressed apoptotic changes leading to cell destruction during an overnight culture. There are some reports highlighting that Tregs require stimulation after thawing to maintain suppressive function *in vitro* [34, 48, 49], which is also consistent with our finding during the re-stimulation and re-expansion experiment of the thawed Tregs.

Since, the impact of cryopreservation on Treg cells is uncertain and there is still lack of proven strategy of cell banking for Treg-based clinical therapies, we decided to test two clinically feasible strategies of cell banking and investigated how cryopreservation affected isolated CD4^+^ cells or expanded Tregs.

We assessed viability with apoptosis, as well as the characteristic Treg phenotype upon cells thawing and compared it to the phenotype before cryopreservation. Our data indicated that although the frequency of necrotic cells was below 10% just after cryopreservation for both, frozen CD4^+^ cells and expanded Tregs, the frequency of cells in the early apoptotic state was much higher (Figure 3). This observation could be explained by the fact that an overnight culture after cryopreservation lead to further decrease in the number of viable cells (Figure 2). Interestingly, the percentage of early apoptotic cells in thawed Tregs was two times higher than in comparison to thawed CD4^+^ cells, suggesting that Tregs are more sensitive to the cryopreservation process. Similar results were noted when Tregs generated from cynomolgus monkeys were frozen and thawed [49]. The high apoptotic rate of thawed Tregs could also explain the above mentioned observation that when Tregs were infused just after thawing, there was no increase in the number of Tregs in the recipients’ peripheral blood [39]. No apoptosis was assessed in this clinical trial and only a viability assay with 7AAD was included in the final Treg product release criteria [39].

Furthermore, upon cryopreservation, we observed instability of Treg markers expression. We noted a significant decrease in the frequency of cells with the phenotype CD4^+^CD25^hi^CD127^-^ and CD4^+^FoxP3^+^ just after cryopreservation, which decreased even more after an overnight culture. This observation could again be associated with the high frequency of observed apoptotic Tregs. During apoptosis, the caspase activation leads to degradation of cellular proteins as well as morphological and physiological changes in cells [50]. Similarly, DMSO used during cryopreservation can activate proteinases that cause cleavage of specific proteins. Such mechanism was suggested as an explanation for the decrease of CD62L expression (crucial for Treg functionality) after CD34^+^ cell thawing [51]. Tregs lacking CD62L have impaired trafficking, resulting in compromised suppressive capabilities [52]. Freezing/thawing of Tregs was also associated with decrease in CD62L expression in mice models [53]. Infusion of thawed Tregs did not protect from GvHD, whereas freshly isolated Treg infusion did [53].

In our study, to overcome Treg cell loss, poor viability and impaired phenotype, we decided to re-stimulate Tregs during subsequent *ex-vivo* expansion. We hoped that such an approach would allow us to not only rescue Treg functionality, but to also restore a sufficient number of cells for clinical application. During our expansion, Treg viability and phenotype were improved and remained stable during expansion. Tregs also met all the release criteria for clinical application with an appropriate fold increase during the expansion, reassuring a sufficient number of cells for clinical application.

In our experience, Tregs isolated from fresh starting material and never submitted to cryopreservation indicate good viability, what could be confirmed by their high proliferation rate and stability of the cell marker expression during the entire expansion period. Interestingly, Tregs, which were initially expanded, then cryopreserved for storage, can resume their proliferative capabilities after thawing during subsequent stimulation and *ex vivo* expansion. The range of fold increase in number of those Treg after expansion was similar comparing to the fold increase in number of Tregs during fresh Treg expansion in the same conditions [28].

Our results indicate that strategy with cryopreservation of expanded Tregs is feasible, but Tregs after thawing can benefit from *ex-vivo* re-stimulation with expansion. This allows for restoration of their properties, the ability to overcome poor recovery and viability as well as recovery from a decline in Treg marker expressions that occurred during cryopreservation.

Furthermore, just after thawing of CD4^+^ cells, we also observed a decrease in the percentage of CD4^+^CD25^hi^CD127^-^ and CD4^+^FoxP3^+^ cells. However, after an overnight culture, the frequency of cells with above phenotypes increased to the percentage before cryopreservation. The decrease in Treg frequency just after cryopreservation is in agreement with previous reports showing that Treg frequency is lower in frozen samples of PBMCs in comparison to fresh ones [54–56]. It is also possible that the observed increase in the percentage of CD4^+^CD25^hi^CD127^-^ and CD4^+^FoxP3^+^ cells might not have been Treg- specific, but related to overall T cell activation after overnight culture since FoxP3 and CD25 markers can also be expressed on activated T cells [57]. Instability of the Treg markers expression could compromise separation of highly pure Tregs from cryopreserved CD4^+^ cells. Decreased viability and progressing apoptosis can also alter cell receptors and affect the results of the sorting. During the cell isolation process, cells are exposed to very stressful conditions with multiple washes, centrifugations, passing through the sample lines in the sorter or magnetic columns, and exposition to antibodies. All of that could additionally alter cell viability, physiology and cell surface receptor expression. Despite these potential drawbacks, we were able to successfully sort Tregs from cryopreserved pre-enriched CD4^+^ cells using FACS. We applied settings of “precision mode”, speed <10,000 events/sec and gate for Tregs, selecting those cells which express CD25 only on the highest level [28]. We believe that such maneuvers allow selecting an extremely pure Treg cell population, preventing contamination with other cells. Such maneuvers can be applied only during FACS, but not when utilizing the immunomagnetic bead separation. Therefore, in our opinion, FACS has a clear advantage over bead selection in Treg processing by improving purity and quality of the final product. During subsequent *ex-vivo* expansion, higher purity of Tregs allowed maintenance of stable CD4^+^CD25^hi^CD127^-^ phenotype and FoxP3 expression of the cell product.

All together, above described strategies to cryopreserve cells are feasible to serve as source material for subsequent Treg isolation and expansion. However, poor recovery and instability of Treg marker expressions upon cryopreservation should be taken in consideration and proper strategies need to be applied to overcome disadvantages to produce a high purity and quality biological cell product.

In conclusion, we tested two strategies for cell banking of Treg-based therapies: 1) cryopreservation of CD4^+^ cells for subsequent Treg isolation and expansion and 2) cryopreservation of expanded Tregs. Our results indicate that cell cryopreservation is associated with induction of apoptosis, leading to poor cell recovery as well as instability of expression of Treg cell markers that compromises cell function and effectiveness of separation techniques. These disadvantages can be overcome by implementing proper cell processing strategies and Treg isolation techniques. In our settings, utilization of FACS for Treg isolation from cryopreserved cells after thawing as well as subsequent *ex-vivo* re-stimulation and re-expansion were effective procedures, resulting in sufficient quality and quantity of human Tregs for clinical applications.

## MATERIALS AND METHODS

### CD4^+^ cells isolation and Treg manufacturing process

CD4^+^ cells and Tregs were isolated according to the previously described process [28]. Briefly, CD4^+^ cells were pre-enriched from leukapheresis product (AllCells, Alameda, CA, USA) obtained from healthy volunteers via immunomagnetic isolation with the use of clinical-grade devices and reagents (CliniMACS^®^, Miltenyi Biotec GmbH, Bergisch Gladbach, Germany). From isolated CD4^+^ cells, Tregs were sorted to the following phenotype: CD4^+^CD25^hi^CD127^-^ and subsequently expanded for 13 days after stimulation with anti-CD3/anti-CD28 beads (MACS® GMP ExpAct Treg Beads, Miltenyi Biotec GmbH, Bergisch Gladbach, Germany) in the presence of interleukin-2 (IL-2, (Proleukin^®^, Novartis Pharmaceuticals Canada Inc., Dorval, Quebec, Canada). During the expansion, quality of Tregs was assessed systematically by measuring expression of key Treg markers (CD4, CD25, CD127, FoxP3) At day 13 Tregs were collected, counted and expansion beads were removed on Clin*ExVivo*™ MPC^®^ magnet (Life Technologies, Dynal Biotech ASA, Norway). Lastly, a sample to assess final Treg quality was taken. All the procedures of Treg isolation and expansion were performed in the cGMP clean room facility class 10,000.

### CD4^+^ and Treg cell freezing and thawing

After a 13-day expansion, isolated CD4^+^ cells and Tregs were frozen in cryoprotectant solution. Briefly, the expanded cells were re-suspended in 100 mls of Plasmalyte A (Baxter Healthcare Corporation, Deerfield, IL, USA) containing 5% Human Serum Albumin (Baxter Healthcare Corporation, Deerfield, IL, USA) at a cell concentration of 500 × 10^6^ /ml. The 100 mls of cells were placed in a transfer bag set (Charter Medical, T3104) followed by the addition of 50 mls of a pre-made chilled (4–6°C) solution consisting of 40 mls of 25% HSA and 10 mls of Plasmalyte A. Such cell solution was chilled for 15 minutes prior to the addition of the final cryoprotectant solution. The 2X cryoprotectant solution comprised of 20 mls of Dimethyl Sulfoxide (DMSO; Bioniche Pharma, Lake Forest, IL, USA) and 30 mls of Plasmalyte A that was also chilled. In the final step prior to initiating the cryopreservation step, the cryoprotectant solution was slowly added to the rest of the solution containing cells and placed in the transfer bag set. Once the cells were mixed well with the cryoprotectant, the 200 mls final volume was divided into four cryobags (Charter Medical CF250). Cryopreservation of cells was carried out in Cryomed, model # 7452 controlled rate freezer using the freezing protocol described in Table 1. Once the cells reached the temperature of -90^0^C they were immediately transferred to the liquid nitrogen storage tank and stored in the vapor phase (–196°C).

**Table 1:** Program used to freeze cells in Cryomed, model # 7452 controlled rate freezer

**Step 1**	Wait at Chamber = 10.0°C
**Step 2**	Wait at Chamber = 0.0°C until Sample = 10.0°C
**Step 3**	Ramp 2.0°C/min. until Sample = –6.0°C
**Step 4**	Hold at Chamber = –20.0°C for 10.0 minutes
**Step 5**	Ramp 15.0°C/min. until Chamber = –60.0°C
**Step 6**	Ramp 15.0°C/min. until Chamber = –45.0°C
**Step 7**	Wait at Chamber = –50.0°C until Sample = –30.0°C
**Step 8**	Ramp 1.0°C/min. until Sample = –60.0°C
**Step 9**	Ramp 5.0°C/min. until Sample = –90.0°C
**Step 10**	End

After over a year of storage in liquid nitrogen vapor phase, bags with cells were thawed in a circulating water bath filled with 0.9% sodium chloride (Baxter Healthcare Corporation, Deerfield, IL, USA) warmed-up to 37°C, until ice crystals disappeared. When thawed, cells were washed once by adding two times the thawed product volume of a solution containing Plasmalyte A and 5% human serum albumin solution (Baxter Healthcare Corporation, Deerfield, IL, USA) to remove DMSO. Following the washing steps, cell pellets were suspended in *X-VIVO*™ 20 medium (Lonza Walkersville, Inc., Walkersville, MD, USA) supplemented with 10% human AB serum (Valley Biomedical Products & Services, Inc., Winchester, VA, USA) and 100 U/ml of IL-2 (Proleukin^®^, Novartis Pharmaceuticals Canada Inc., Dorval, Quebec, Canada). Cells were cultured overnight at a final concentration 1 × 10^6^ cells per 1 ml of culture medium.

The next day, cells were collected, washed again in the same fashion as after cryopreservation, counted and processed as follows:

1) CD4^+^ cells were stained with fluorochrome-conjugated monoclonal antibodies: CD4 PerCP, CD25 APC, CD127 PE (BDBiosciences, San Jose, CA, USA) and submitted for Tregs (CD4^+^CD25^hi^CD127^-^ cells) sorting with FACSAria III cell sorter (BDBiosciences, San Jose, CA, USA).
2) Treg cells were stimulated with anti-CD3/anti-CD28 beads (MACS^®^ GMP ExpAct Treg Beads, Miltenyi Biotec GmbH, Bergisch Gladbach, Germany) at 1:1 bead to cell ratio for *ex-vivo* expansion.

Tregs sorted from thawed CD4^+^ cells (1) and retrieved after thawing previously expanded Tregs (2) were expanded for 13 days accordingly to the previously described protocol [28].

### Post-thawing recovery

Immediately after cell thawing, washing and re-suspension, the samples were taken for total cell count. Cell counting was done on hemocytomer using the trypan blue exclusion assay (improved Neubauer chamber, Hausser Scientific, Horsham, PA, USA). Cell enumeration was also performed following an overnight culture, collection, washing and re-suspension in the Culture Medium.

### Viability with apoptosis measurements after cell thawing

Immediately after thawing, samples of 2 × 10^5^ cells were taken, washed in PBS, re-suspended in 100 μl of Annexin-V binding buffer (BD Biosciences, San Jose, CA, USA) then stained with Annexin-V FITC (Life Technologies, Eugene, OR, USA) and 7 amino-actinomycin (7AAD, ViaProbe™, BD Biosciences, San Jose, CA, USA). After 15 minutes of incubation, 400 μl of Annexin-V binding buffer was added to the sample to dilute dyes. The samples were immediately transferred to the flow cytometer and analyzed. Annexin-V^+^7AAD^-^ cells were considered as early apoptotic and Annexin-V^+^7AAD^+^ as late apoptotic/necrotic.

### Treg cell phenotyping

Treg cell phenotype was analyzed immediately after thawing, after subsequent overnight culture and during Treg expansion (on days: 0, 7, 13). To measure Treg phenotype, a sample with 2 × 10^5^ cells was taken and processed accordingly to the manufacturer instruction for the FoxP3/Transcription Factor Staining Buffer Set (eBioscience, Inc., San Diego, CA, USA). Subsequently, a sample was stained with the following antibodies: FoxP3 FITC (eBioscience, Inc., San Diego, CA, USA), CD4 PerCP, CD25 APC, CD127 PE (BDBiosciences, San Jose, CA, USA). Data for cell phenotyping was acquired by flow cytometry.

### Suppression of proliferation assay

Treg function was checked after the expansion in suppression of proliferation assay. Briefly, Teffectors were stained with 5μM carboxyfluorescein diacetate succinimidyl ester (CFSE) (Molecular Probes, Inc., Invitrogen, Eugene, OR, USA) and plated with expanded Tregs in different proportions (Treg:Teffector – 1:1, 1:2, 1:8). Cells were stimulated with anti-CD3, anti-CD28-coated magnetic beads (LifeTechnologies, USA) at 1:1 bead to cell ratio and co-cultured for 4 days in the presence of IL-2 at a concentration 100 U/ml in culture medium. Assay was prepared in triplicate. After co-culture, CFSE dye dilution was measured by flow cytometry.

### Flow cytometry

Minimum of 10,000 events were acquired per analyzed sample on LSRFortessa™ or LSR II Cell Analyzers (BDBiosciences, San Jose, CA, USA) and subsequently analyzed on FlowJo^®^ software (FlowJo, LLC., Ashland, OR, USA). Isotype or Fluorescence Minus One (FMO) controls were used for gate settings.

### Release criteria of the final Treg product

Tregs at the end of the expansion were tested to determine if the cells passed the following release criteria for clinical application:

(1) sterility (Gram stain and 14-day anaerobic, aerobic and fungal culture) – to exclude presence of bacteria and fungal growth; (2) mycoplasma test (MycoAlert Mycoplasma Assay, Lonza Walkersville Inc., Walkersville, MD, USA) negative when < 0.8; (3) endotoxin (EndoSafe^®^ PTS™ Endotoxin System, Charles River, Charleston, SC, USA) - product accepted for infusion when < 5 EU/kg; (4) determination of residual expansion beads – maximum allowed < 100 beads per 3 × 10^6^ cell, (5) viability with 7 AminoActinomycin D (7AAD) – acceptable value > 75%; (6) % of FoxP3^+^ cells – acceptable value > 60%, (7) % of CD4^+^ cells – acceptable value > 90%, (8) % of CD8^+^ cells acceptable value < 5%. As described in the previously published protocol [28], the release criteria were established based on FDA requirements and were utilized in testing the final Treg product in clinical trials conducted in the USA [39, 41, 42]. Viability with 7AAD, % of FoxP3^+^, CD4^+^ and CD8^+^ cells was determined by flow cytometry. Most of the assays for the release criteria were validated and performed by the College of American Pathologists (CAP)- certified laboratories. Determination of residual expansion beads was performed “in house” after assay validation by our team. Five ml of Treg suspension was retrieved from the infusion bag after bead removal and Tregs were tested to ensure fulfillment of the release criteria.

### Data analysis

Data were analyzed with the GraphPad Prism v5.0 software (GraphPad Software, Inc., San Diego, CA, USA). Data are expressed as mean +/– SD. Two-tailed paired *t*-test was used to compare values after thawing to values prior to cryopreservation. Statistical significance was accepted when *p* < 0.05.

### Ethical considerations

We obtained de-identified human cell leukapheresis product for all the experiments from the commercial vendor AllCells, Alameda, CA, USA, therefore the study was exempt from the Institute Review Board (IRB) review. In addition, the vendor certified that the product was obtained from volunteers participating in an IRB or Human Subject Committee approved donor program and had current IRB approval. Vendor also certified that all donors provided informed consent for use of the cells in any research study.
